# Treatment of Invasive Candidiasis: A Narrative Review

**DOI:** 10.3390/jof4030097

**Published:** 2018-08-16

**Authors:** Ronen Ben-Ami

**Affiliations:** Infectious Diseases Unit, Tel Aviv Sourasky Medical Center, and the Sackler Faculty of Medicine, Tel Aviv University, Tel Aviv 64239, Israel; ronenba@tlvmc.gov.il; Tel.: +972-3-6974347; Fax: +972-3-6974996

**Keywords:** *Candida*, invasive candidiasis, candidemia, fluconazole, echinocandin, amphotericin B

## Abstract

Invasive candidiasis occurs frequently in hospitalized patients, and is associated with high mortality rates due to delays in recognition and initiation of appropriate antifungals. Management of invasive candidiasis must take into account multiple host, pathogen, and drug-related factors, including the site of infection, host immune status, severity of sepsis, resistance and tolerance to antifungal agents, biofilm formation, and pharmacokinetic/pharmacodynamic considerations. Recent treatment directives have been shaped by the widespread introduction of echinocandins, highly potent and safe antifungals, into clinical use, as well as important changes in drug susceptibility patterns and the emergence of known and novel drug-resistant *Candida* species. Advances in molecular diagnostics have the potential to guide early targeted treatment of high-risk patients.

## 1. Background

*Candida* species are frequent colonizers of the human alimentary tract and skin that have emerged as important nosocomial pathogens, in tandem with advances in modern medical therapeutics [1,2]. Notable risk factors for invasive candidiasis include exposure to broad-spectrum antibiotics and cancer chemotherapy, advanced care of premature neonates, major abdominal surgery, organ transplantation, prolonged stay in an intensive care setting, implanted medical devices such as vascular catheters and prosthetic heart valves, and parenteral feeding. Invasive candidiasis, a term that encompasses the overlapping syndromes of deep-seated candidiasis and candidemia, is the most frequent mycotic disease in hospitalized patients. In an often cited nationwide survey, *Candida* species were found to be the fourth most frequent causes of bloodstream infection in hospitalized patients in the US, and were associated with the highest mortality rate among leading bloodstream isolates [2]. In point prevalence studies, *Candida* species account for about 1 in 5 nosocomial bloodstream infections [1,3]. Deep-seated candidiasis, arising from direct inoculation or hematogenous dissemination of *Candida* into normally sterile body sites, is often difficult to diagnose and may affect a population as large as that of candidemia [4,5,6]. The global incidence of candidiasis has been estimated at 750,000 cases annually (2.1 to 21 cases per 100,000 population) [4], with an associated crude mortality rate in excess of 40% and an associated expenditure of ~$46,000 per case [7].

Although *Candida albicans* is the most common cause of invasive candidiasis, infections due to non-*albicans Candida* species account for an increasing proportion of cases. Specifically, *C. glabrata* has become an important pathogen in North America, Europe (with the exception of Spain), and Australia, whereas *C. parapsilosis* is the dominant non-*albicans* species in South America, Japan, and Spain [8,9,10]. The shift from *C. albicans*, which is overwhelmingly susceptible to all systemic antifungals, to species that are more frequently resistant or tolerant to fluconazole, such as *C. glabrata* and *C. krusei*, has impacted treatment recommendations [11,12,13]. *Candida* strains with acquired resistance to echinocandins, specifically *C. glabrata*, while still generally infrequent, are increasing in incidence in some hospitals [10,14]. *C. auris*, a novel *Candida* species first identified in 2009, has emerged since 2013 as a cause of nosocomial outbreaks in multiple countries across 5 continents. *C. auris* presents a serious challenge to healthcare systems due to its frequent resistance to multiple systemic antifungals and disinfectants and its capacity for rapid spread within healthcare facilities [15,16].

## 2. Systemic Antifungal Drugs for Invasive Candidiasis

The armamentarium of drugs for the treatment of candidiasis currently comprises three major drug classes: the polyenes, azoles, and echinocandins. In addition, flucytosine, a pyrimidine analogue, has specific roles as an adjunct in the treatment of central nervous system candidiasis and *Candida* endocarditis [17,18].

### 2.1. Echinocandins

The echinocandins are cyclic lipopeptides that inhibit the transmembrane glucan synthase complex (Fks1), a biosynthetic enzyme that produces 1,3-β-d-glucan, the major fungal cell-wall carbohydrate. Disruption of 1,3-β-d-glucan synthesis damages cellular integrity and leads eventually to cell rupture and death. All three licensed echinocandins (caspofungin, micafungin, and anidulafungin) are approved for the treatment of invasive candidiasis. These drugs may only be given intravenously. Caspofungin and micafungin are metabolized in the liver, whereas anidulafungin is slowly degraded in plasma due to chemical opening of its ring structure.

The echinocandins are fungicidal in vitro against a broad range of *Candida* species, including azole resistant or tolerant species. *C. parapsilosis* and *C. guilliermondii* are intrinsically less susceptible to echinocandins, reflecting naturally occurring polymorphisms in the *FKS1* hotspot regions of these species [19].

The echinocandins are remarkably safe, even at higher than standard doses. Rapid infusion has been associated with flushing, dyspnea, hypotension, and urticaria, attributed to histamine release. These drugs should therefore be infused over 1 h. Cardiotoxicity is a rare and severe side effect that has been observed when echinocandins are administered through a central venous catheter. This phenomenon may be less frequent with anidulafungin than with caspofungin and micafungin. No significant hepatotoxicity or nephrotoxicity have been observed.

Drug-drug interactions are relatively few with echinocandins. Caspofungin clearance is increased by inducers of liver CYP450 enzymes, such as rifampicin, efavirenz and carbamazepine, requiring a caspofungin dose increase from 50 mg/day to 70 mg/day if co-administered with those drugs. Caspofungin exposure is increased if co-administered with cyclosporin. Caspofungin decreases tacrolimus exposure, requiring drug level monitoring and dose adjustment. Micafungin and anidulafungin are not modulators or substrates of CYP450 enzymes, and were not found to have important drug-drug interactions.

#### Clinical Trials

Prospective studies comparing echinocandins with amphotericin B for the treatment of candidiasis have shown similar efficacy of caspofungin and amphotericin B deoxycholate in the intention to treat analysis but superiority of caspofungin in the per-protocol analysis [20], and non-inferiority of micafungin versus liposomal amphotericin B [21]. In both trials, echinocandins were associated with fewer adverse events, including serious events leading to drug discontinuation. Anidulafungin was associated with a higher treatment success rate than was fluconazole, but mortality rates were not significantly different [22]. Interestingly, the superiority of anidulafungin was most significant for patients with *C. albicans* infection [22], indicating that drug susceptibility per-se could not account for the differences in treatment outcomes. Finally, two trials compared different echinocandin drugs and doses. A 3-arm study compared micafungin 100-mg daily, micafungin 150-mg daily and caspofungin (70 mg followed by 50 mg daily). Micafungin at both doses was non-inferior to caspofungin, whereas the median time to culture negativity was 1 day longer for the micafungin 150-mg dose versus the micafungin 100-mg dose [23]. A study comparing a triple daily dose of caspofungin (150-mg daily) versus standard 50-mg daily dosing found no advantage for the higher dose [24]. Taken together, these studies show that higher than standard doses of echinocandins, while generally well tolerated, do not improve patient outcomes in most clinical scenarios.

### 2.2. Azoles

Azoles inhibit *Candida* growth by binding to and inhibiting lanosterol 14α demethylase, a cytochrome P450 enzyme encoded by the *ERG11* gene [25]. Lanosterol 14α demethylase inhibition causes depletion of ergosterol, the main plasma membrane sterol in fungi, and accumulation of toxic C14α methylsterols. The antifungal effect is fungistatic rather than fungicidal.

Fluconazole is the principle azole drug used in the treatment of candidiasis. Fluconazole has excellent bioavailability and may be given either intravenously or orally at identical doses. The main adverse effect of fluconazole is hepatotoxicity. In addition, fluconazole is a cytochrome P450 inhibitor and may interact with drugs that undergo metabolism via that route.

#### Clinical Trials

In randomized controlled clinical trials of nonneutropenic patients with candidemia, outcomes were similar for patients treated with fluconazole versus amphotericin B, but toxicity was more frequent with amphotericin B [26,27]. Treatment failure occurred most frequently with *C. albicans* [26]. A study comparing fluconazole monotherapy with fluconazole combined with amphotericin B found a higher overall treatment success rate and a lower rate of failure to clear the bloodstream with the combination regimen [28]. Patients with the lowest and highest APACHE II scores benefited the least from combination therapy, suggesting that fluconazole monotherapy is adequate for candidemic patients with mild disease, whereas for the sickest patients, failure rates are high regardless of treatment choice [28].

Voriconazole, a broad spectrum triazole active against filamentous fungi as well as *Candida* species, was as effective as amphotericin B followed by fluconazole for the treatment of candidemia [29]. Voriconazole is associated with more adverse events and drug-drug interactions than fluconazole, and offers few advantages over fluconazole in the treatment of candidiasis. In practice, this drug may be useful in the treatment of infections with the intrinsically fluconazole-resistant species *C. krusei*.

### 2.3. Amphotericin B

The polyene amphotericin B is a lipophilic molecule that binds to ergosterol within the fungal cytoplasmic membrane. Amphotericin B self-assembles into oligomers that insert into the cytoplasmic membrane, forming aqueous pores that cause ion leakage and rapid cell lysis. Amphotericin B is formulated with deoxycholate or in one of three lipid formulations (liposomal amphotericin B, amphotericin B lipid complex and amphotericin B colloidal dispersion). The specific formulation has no effect on the drug’s spectrum of activity, but does alter its distribution to different organs.

Once the mainstay of antifungal treatment, amphotericin B has been demoted to a second-line treatment of invasive candidiasis due to its toxicity. The nephrotoxicity caused by amphotericin B may be divided into early reversible azotemia due to afferent arterial vasoconstriction, and late kidney damage due to nephron loss and tubular basement membrane damage. The latter is irreversible and correlates with the total dose of amphotericin B infused. Overall, acute kidney injury occurs in 30% of adults treated with amphotericin B, and is associated with significantly increased mortality rates, length of hospital stay, and costs [30]. Early infusion reactions manifesting as chills and fever are common, and tend to lessen with subsequent drug infusions. Lipid amphotericin B formulations are less nephrotoxic, and liposomal amphotericin B and amphotericin B lipid complex are associated with fewer acute infusion reactions than amphotericin B deoxycholate.

## 3. Practical Aspects of Antifungal Susceptibility Testing

In vitro susceptibility testing is performed using broth microdilution (Clinical and Laboratory Standards Institute (CLSI) or European Committee on Antimicrobial Susceptibility Testing (EUCAST) methodology) [31,32]. Less labor-intense methods employed by clinical microbiology laboratories include Etest (bioMerieux, Marcy l’Etoile, France), disk diffusion, Sensititre Yeast One (TREK Diagnostic Systems, Thermo Fisher Scientific Inc., Waltham, MA, USA), and various semiautomated methods such as the Vitek system (bioMerieux).

### 3.1. Echinocandin Susceptibility Testing

Echinocandin minimal inhibitory concentrations (MICs) are read on broth microdilution assays as the drug concentration that produces significant (~50%) reduction in turbidity after 24 h [31]. Caspofungin has been found to be unreliable for MIC determination due to high interlaboratory variability [33,34,35,36]. Therefore, anidulafungin and micafungin are used for susceptibility testing, and caspofungin susceptibility may be inferred from these results.

A paradoxical (or eagle) echinocandin effect has been observed in which increased *Candida* growth is detected at higher than MIC drug concentrations. This effect is more pronounced with caspofungin than with other echinocandins, is unrelated to MIC, appears to be most common with *C. albicans* and *C. dubliniensis*, and is strikingly absent in *C. glabrata* [37,38,39]. The paradoxical effect is abolished by testing in 50% human serum and in the presence of the chitin synthase inhibitor nikkomycin Z and calcineurin inhibitors, suggesting that cell-wall stress response pathways are involved [39]. The clinical relevance of the paradoxical effect is incompletely understood. No correlation has been found with survival rates in clinical trials. However, an animal model of peritoneal *C. tropicalis* infection using a strain showing paradoxical growth demonstrated impaired fungal clearance with high caspofungin concentrations [40], suggesting that this phenomenon may contribute to persistence and relapsed infection in vivo.

### 3.2. Resistance to Echinocandins

Clinically significant acquired resistance of *Candida* species to echinocandins has, thus far, always been associated with single amino acid substitutions arising from mutations within conserved (“hot spot”) sequences of the gene *FKS1*, and in *C. glabrata FKS2*, which encode for glucan synthase. In multivariate analyses, *FKS* mutations, rather than echinocandin MIC, were the only significant predictor of clinical echinocandin treatment failure [41]. Therefore, molecular detection of *FKS* mutations may be the most accurate way of classifying echinocandin resistant isolates. As there are currently no commercially available assays for *FKS* genotyping, molecular resistance testing is limited to reference laboratories.

Acquired echinocandin resistance has been described in all major *Candida* species, but is most frequently encountered in *C. glabrata*, where an association with echinocandin treatment failure has been described [14,42]. Echinocandin-resistant *Candida* isolates have impaired fitness as compared with isogenic *FKS* wild-type strains [43,44]. This fitness cost has been demonstrated using in vivo virulence and competitive growth experiments, and is associated with morphological and functional changes, namely slower growth rate, a thick cell wall with reduced glucan and increased chitin content, and impaired filamentation of the *FKS* mutants [43]. Perhaps related to their reduced fitness, *Candida* species carrying *FKS* hotspot mutations are almost invariably recovered from patients with extensive recent exposure to echinocandins, and usually after more than 3 weeks of treatment [45].

### 3.3. Fluconazole Susceptibility Testing

Susceptibility testing to fluconazole using CLSI or EUCAST broth microdilution methodology produces MIC values that correlate reasonably well with the likelihood of successful treatment outcomes [46,47]. The MIC is read at 24 or 48 h as the minimal drug concentration resulting in significant (~50%) reduction in growth [31]. Pfaller et al. [46] set forth fluconazole clinical breakpoints that met the so-called “90–60” rule, whereby treatment success is achieved for ~90% of susceptible isolates and ~60% of non-susceptible isolates. In 2012, these breakpoints were revised to account for species-specific differences in drug response, as well as to enhance sensitivity to detect the emergence of fluconazole-resistant strains [47]. Breakpoints for *C. albicans*, *C. tropicalis*, and *C. parapsilosis* were lowered, so that MICs over 4 mg/L are considered resistant. For *C. glabrata*, an inherently fluconazole-tolerant species, an MIC greater than 32 mg/L is considered resistant, whereas an MIC of 32 mg/L or less is considered susceptible dose-dependent (S-DD), a term indicating that maximal drug dosage is required for optimal treatment.

### 3.4. Resistance to Fluconazole

Unlike the canonical resistance mechanism to echinocandins, acquired resistance to fluconazole may arise by various mechanisms that differ among the *Candida* species. Drug efflux is the most frequent mechanism, and is mediated by transmembrane transporter molecules of either the ATP-binding cassette (ABC) or major facilitator (MFS) superfamilies [48,49]. Resistance may also arise from mutations in the *C. albicans ERG11* coding sequence, resulting in reduced drug-to-target affinity, and gain-of-function mutations in the gene encoding transcription factor *UPC2* that lead to enhanced *ERG11* expression [50]. Acquired fluconazole resistance may arise in any *Candida* species following prolonged exposure to the drug, as previously shown for AIDS patients with oropharyngeal and esophageal candidiasis [49]. Of the major pathogenic *Candida* species, *C. glabrata* is the most prone to acquire fluconazole resistance, usually associated with upregulation of the ABC efflux pumps *CDR1* and *CDR2* [51,52]. A gain-of-function mutation in the gene encoding for transcription factor *PDR1* leads to coordinated upregulation of multiple ABC-type drug transporters [53,54,55,56,57]. Contrary to echinocandin resistance, fluconazole-resistance does not incur a significant fitness cost, and may in fact be associated with enhanced virulence [55]. *C. krusei* is intrinsically resistant to fluconazole because of *ERG11* polymorphism, but is usually susceptible to other azoles such as voriconazole and posaconazole. *C. auris* is intrinsically resistant to fluconazole, and cannot reliably be treated with other azoles [16,58,59].

### 3.5. Amphotericin B Susceptibility Testing

Amphotericin B MIC values are determined as the drug concentration that produces complete or near-complete reduction in growth at 24 h or 48 h. Correlation between amphotericin B MIC values and clinical response has not been consistently established [60,61]. Based on pharmacokinetic data and epidemiological cut-off values, *C. albicans*, *C. tropicalis*, *C. parapsilosis*, *C. glabrata*, and *C. krusei* isolates with MIC > 1 mg/L are assumed to be resistant to amphotericin B [31,62].

### 3.6. Resistance to Amphotericin B

Primary resistance to amphotericin B is uncommon. Two related species, *C. lusitaniae* and *C. auris*, display higher MICs for amphotericin B than other species. Acquired resistance is also rare, and involves reduced ergosterol biosynthesis and the production of alternative sterols to which amphotericin B has low affinity. *Candida* strains resistant to amphotericin B were found to harbor mutations in *ERG2*, *ERG3*, *ERG5*, and *ERG11*, and were co-resistant to fluconazole [63,64,65,66].

### 3.7. Development of Antifungal Resistance in C. glabrata

*C. glabrata* is uniquely predisposed to become resistant to antifungals during treatment. Recent work has revealed both genetic and epigenetic mechanisms underpinning this propensity. Specific mutations in the DNA mismatch repair gene *MSH2* lead to a hyper-mutable phenotype and were found to be highly prevalent in clinical *C. glabrata* isolates [67]. *MSH2* deletion significantly increases breakthrough infection and the emergence of resistance to all systemic antifungal drug classes in *in vitro* and animal experimental systems [67]. Heteroresistance, specifically to fluconazole, was also shown to be highly frequent among clinical *C. glabrata* strains, and is associated with failure of fluconazole to clear *C. glabrata* from tissues of experimentally infected immunocompetent mice [68]. Heteroresistance implies cell-to-cell variation in drug response within a genetically homogeneous population, and may lead to persistence of a sub-population of cells exposed to high drug concentrations [69]. Heteroresistance to fluconazole is associated with up-regulation of ABC-type drug transporters, and may be detected and quantified using population analysis profiling [68], a method not routinely used by clinical microbiological laboratories.

## 4. Treatment Strategies

The goals of treatment of patients with invasive candidiasis are to prevent three types of adverse outcomes: 1. Death, usually within the first 7 days after onset of infection; 2. Late complications from metastatic and persistent deep-seated infection; and 3. Emergence of drug-resistant *Candida* strains.

### 4.1. Early Initiation of Antifungal Therapy

There is extensive clinical evidence that delayed initiation of appropriate antifungal treatment is associated with increased mortality in patients with invasive candidiasis [70,71,72]. This association is particularly pronounced for patients with candidemia and septic shock, for whom initiation of antifungal treatment more than 24 h after the onset of shock is associated with a mortality rate of nearly 100% [73]. However, blood culture, the standard tool for diagnosis of invasive candidiasis, has suboptimal sensitivity, especially for those patients with deep-seated candidiasis [74] and for patients infected with *C. glabrata* [75]. Moreover, the median time to blood culture positivity is 2–3 days, and may be as long as 8 days. Again, median time to detection is longer for *C. glabrata* versus other species [75]. Consequently, blood cultures are too insensitive and delayed in their efficacy to serve as triggers for early appropriate antifungal treatment. Strategies to identify patients who would benefit from treatment of incipient invasive candidiasis have included risk scores and biomarkers.

Risk factors for invasive candidiasis have been incorporated into a number of clinical prediction rules. The best known of these, the *Candida* score, assigns points for total parenteral nutrition, surgery, multifocal *Candida* colonization (1 point each), and severe sepsis (2 points) [76]. A total score of 3 or more has 81% sensitivity and 74% specificity for the diagnosis of invasive candidiasis, and a 7.7-fold greater risk of candidiasis [76]. The *Candida* score is simple, accessible, and inexpensive. Real-life use of this prediction rule in the intensive care setting reveals a high negative predictive value (80% to 97%) but modest positive predictive value (57% to 70%) [77,78]. The positive predictive value increases with the numeric value of the *Candida* score [79].

A number of non-culture based biomarkers have been utilized to guide therapy of invasive candidiasis. The performance of all biomarkers is improved when multiple test results are analyzed sequentially over time. (1,3)-β-d-glucan is a cell wall component of *Candida* species and other pathogenic fungi. A meta-analysis of 16 trials found a pooled sensitivity of 76% and specificity of 85% [80]; false positive results are attributed to the ubiquity of glucans in the environment. An assay that detects both mannan and anti-mannan IgG (Platelia, Bio-rad, Hercules, CA, USA) has a combined sensitivity and specificity of 83% and 86%, respectively [81]. Finally, PCR assays that detect *Candida* DNA in full blood or serum are both sensitive and specific [82]. However, until recently, there have been no commercially available *Candida* PCR assays that underwent extensive clinical validation. The T2Candida assay (T2Biosystems, Lexington, MA, USA) combines real time PCR with amplicon-induced agglomeration of supermagnetic nanoparticles and T2 magnetic resonance detection. In a multicenter study of patients with candidemia, the sensitivity of T2Candida was double that of conventional blood cultures when both were tested on follow-up blood samples [83]. Patients who had received antifungal treatment were more likely to have negative blood cultures and positive T2Candida results. Overall, when used in the appropriate clinical setting, biomarkers offer opportunities for early detection and treatment of invasive candidiasis. T2Candida represents an important advance as the first commercially available, fully-automated rapid assay that specifically detects *Candida* species.

### 4.2. Choice of Antifungal Agent

**Figure 1:**
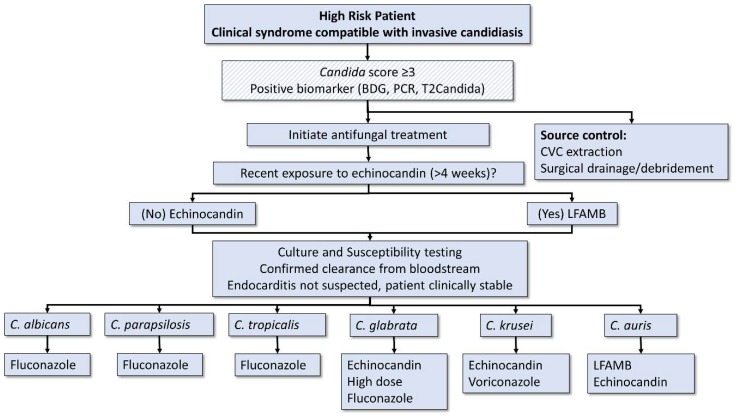
Treatment algorithm for patients with suspected or confirmed invasive candidiasis. Scored fill pattern indicates an optional step. For considerations regarding timing of CVC removal, see text. Echinocandin indicates caspofungin, micafungin or anidulafungin. BDG: (1,3)-β-d-glucan; LFAMB: lipid formulation of amphotericin B; CVC: central venous catheter.

For most forms of invasive candidiasis, echinocandins are recommended as front-line agents, for the following reasons: 1. Broad spectrum activity against the majority of clinically important *Candida* species; 2. Excellent safety profile; and 3. Clinical superiority when compared to other systemic antifungals. A patient-level meta-analysis of 7 clinical trials with 1915 patients found that treatment with an echinocandin was associated with decreased mortality [84]. Lipid formulations of amphotericin B are associated with similar rates of treatment success as echinocandins but higher rates of nephrotoxicity [21]. Their use should be considered for patients with significant (>4 weeks) recent exposure to echinocandins, for whom acquired resistance to this class is a concern (Figure 1) [42].

### 4.3. Species-Specific Considerations

#### 4.3.1. *C. glabrata*

Treatment of *C. glabrata* with fluconazole remains an area of clinical uncertainty. Not only does *C. glabrata* have higher median MICs of fluconazole compared with other common *Candida* species, but treatment failure and the emergence of fluconazole-resistant strains occur frequently during treatment of initially susceptible isolates [52]. In addition, as noted, heteroresistance to fluconazole is frequent in clinical *C. glabrata* isolates [68]. With these considerations in mind, treatment of invasive *C. glabrata* infection with fluconazole should be reserved for cases where: 1. Bloodstream infection has been cleared with a fungicidal agent, such as an echinocandin or amphotericin B formulation; 2. The patient is clinically stable, infected catheters have been removed and deep-seated foci of undrained infection have been ruled out; and 3. The isolate is fluconazole non-resistant. Fluconazole should always be used at maximal doses (i.e., 12 mg per kg daily) in such cases.

#### 4.3.2. *C. parapsilosis*

*C. parapsilosis* is intrinsically less susceptible to echinocandins than other *Candida* species; however, there are conflicting epidemiological and clinical data regarding the significance of this observation. Recent exposure to an echinocandin was found to significantly increase the risk of infection with *C. parapsilosis* [85]. Indeed, the rising incidence of *C. parapsilosis* in some medical centers has been linked to increased echinocandin use [86,87]. Clinical trials have shown numerically more cases of treatment failure for *C. parapsilosis* with anidulafungin versus fluconazole [22] and with caspofungin versus amphotericin B [20], although these differences were not statistically significant. In a trial comparing two dosing regimens of caspofungin, the response rate was non-significantly lower for *C. parapsilosis* in the standard dose regimen (50 mg/day) versus the high-dose regimen (150 mg/day) (61% versus 91%, respectively) [24]. These trials were not designed or able to detect differences in the subgroup of patients with *C. parapsilosis* infection. In contrast, observational clinical data did not show an association between echinocandin treatment of *C. parapsilosis* bloodstream and treatment failure [88]. Echinocandins remain the preferred treatment of patients with *C. parapsilosis* infection, but alternative agents should be considered in patients with persistent infection while receiving an echinocandin.

#### 4.3.3. *C. auris*

Optimal drug treatment of *C. auris* has not yet been defined. For most clinical isolates, MICs of echinocandins are in the susceptible range. Echinocandins are currently recommended by the US centers for disease control as empiric treatment of *C. auris* infections [89]. However, there are no clinical data to support this recommendation, and there is evidence that *C. auris* is tolerant to echinocandins. Exposure to echinocandins was consistently found to be a risk factor for infection with *C. auris*, and breakthrough infection on echinocandins has been noted [16,90,91]. Preliminary *in vitro* and animal data support the use of liposomal amphotericin B, alone or in combination with flucytosine, for patients with known or suspected *C. auris* infection [92].

### 4.4. Treatment Duration and De-Escalation

Two weeks of antifungal treatment seem to be sufficient to prevent late complications from clinically important metastatic foci of infection in the vast majority of cases [93]. Blood cultures should be repeated every 1–2 days after treatment is initiated to document clearance of *Candida* from the bloodstream. In general, once the patient is clinically stable, repeat blood cultures are negative and the *Candida* isolate is found to be sensitive to fluconazole, treatment may be safely switched to fluconazole. This strategy was shown to be safe and feasible for multiple *Candida* species, including *C. glabrata* [94]. De-escalation to oral fluconazole may reduce exposure to echinocandins, limit the emergence of resistance to this important drug class, shorten hospital stays, and limit costs.

## 5. Source Control

The majority of patients with candidemia have indwelling central venous catheters (CVC) when the diagnostic blood culture is obtained [2,95], but differentiating between CVC- and non-CVC-related candidemia is not always straightforward. *C. parapsilosis* is particularly frequent as a cause of CVC infection, and neutropenic patients are relatively more likely than non-neutropenic patients to have *Candida* bloodstream infection that originates in the gut [88]. For individual patients, however, such distinctions lack sufficient predictive power. There is compelling evidence that CVC removal is associated with higher rates of treatment success and lower mortality rates as compared with CVC retention [73,84,88,96,97]. Despite contradictory data from a post-hoc analysis of 2 clinical trials [98], it is generally accepted that indwelling CVCs should be removed as early as possible in all patients with candidemia [11,12,84,96]. CVCs should be urgently removed in patients with septic shock [73]. For clinically stable patients for whom immediate CVC removal presents significant difficulties, for example due to limited vascular access or coagulopathy, establishing a diagnosis of CVC infection may be of importance.

As for bacterial bloodstream infection, differential time to positivity, i.e., the time difference between detection of growth in blood drawn from the CVC lumen to peripherally drawn blood, is a readily-available metric that may be used to predict the likelihood of CVC infection [99]. A differential time to positivity of greater than 2 h is associated with *Candida* CVC infection, but is not useful in cases of *C. glabrata* bloodstream infection [99]. Peripheral blood time to positivity alone may also be of value, as a value greater than 30 h is associated with a significantly lower likelihood of CVC infection [100]. For the slow growing *C. glabrata*, a breakpoint of 48 h may be used [101].

Catheter lock therapy has been used to salvage long-term CVCs infected with low-pathogenicity microorganisms [102]. There is limited clinical experience with the use of this method to eradicate *Candida* species from difficult to extract CVCs [103]. Ethanol, echinocandins and amphotericin B have been used in lock solutions, with anecdotal success [103].

Management of other foci of deep-seated infection is equally important. Intra-abdominal candidiasis tends to progress to abscesses that are persistent and recalcitrant, and if undrained may predispose for the development of drug-resistance [5,104]. Echinocandins in particular may achieve subinhibitory concentrations within abdominal abscesses, underscoring the importance of surgical drainage and debridement.

## 6. Echocardiography

Endocarditis is a rare but devastating complication of candidemia. In an observational single center cohort study, the authors reported findings suggestive of endocarditis in 2.9% of patients who underwent transthoracic echocardiography (TTE) and 11.4% of patients who underwent transesophageal echocardiography (TEE) [105]. Importantly, endocarditis was clinically unsuspected in a third of the cases, prompting a recommendation for TEE in all patients with candidemia [11]. However, other groups have reported much lower incidence rates of endocarditis, suggesting that a more selective strategy may be reasonable [12]. Patients at risk of endocarditis include those with prosthetic heart valves, injectable drug users, patients with prolonged or non-resolving candidemia, and those with new onset of heart failure or embolic phenomena. Further work is needed to calculate the cost-benefit ratio of TEE and to better define subgroups of patients with candidemia who would benefit from an exhaustive search for endocarditis.

## 7. Ocular Complications

Metastatic spread of *Candida* species to the eyes occurs in 9% to 16% of patients with candidemia [106,107], and may lead to loss of vision. Ocular infection initially manifests as chorioretinitis, and if untreated may extend into the vitreous to cause vitritis (endogenous endophthalmitis). Visual outcome is associated with involvement of the retinal macula and the extent of vitritis at the time of diagnosis. All patients with candidemia should have a retinal examination performed by an experienced ophthalmologist [12]. Clinical guidelines recommend retinal examination within the first week of therapy [12]. However, as many as 18% of ocular lesions were only detected on follow-up examination in one study [106], suggesting that it may be prudent to perform an additional retinoscopy during the second week of therapy, especially in patients with persistent candidemia. Because the chorioretinal layer is highly vascular, treatment of chorioretinitis with any of the systemic antifungal drugs should be effective. However, because subclinical vitritis may be present, treatment with antifungals that achieve therapeutic concentrations within the vitreous is preferred. Treatment with fluconazole is recommended if susceptibility to this drug has been established [108]. For fluconazole-resistant isolates, liposomal amphotericin B is preferred over echinocandins, which achieve suboptimal vitreous concentrations [109,110]. Detection of lesions near the macula or vitritis necessitate intravitreal injection of antifungals (100 µL solution of amphotericin B deoxycholate or voriconazole), and in some cases, vitrectomy. Treatment should be extended for at least 4 to 6 weeks with ophthalmological monitoring to assess clearance of the ocular infection.

## 8. Novel Drugs for Invasive Candidiasis

A number of exciting new *Candida*-active compounds are currently in various stages of drug development and clinical trials. These include agents with potent activity against challenging *Candida* species, such as echinocandin- and azole-resistant *C. glabrata* and multidrug-resistant *C. auris*.

Ibrexafungerp (SCY-078, Scynexis, Jersey City, NJ, USA) is a first-in-class oral glucan synthase inhibitor that has shown fungicidal activity against a wide range of *Candida* species in in vitro assays and animal models [111]. Importantly, SCY-078 appears to be active against echinocandin-resistant and azole-resistant *Candida* strains, including *C. auris* [111,112,113]. *C. glabrata* strains with acquired resistance to SCY-078 have already been described, harboring partially novel FKS mutations [114]. Results of a phase 2 trial evaluating SCY-078 as step-down treatment following micafungin are awaited (NCT02244606). SCY-078 is currently being assessed in a single-arm phase 3 trial for the treatment of *C. auris* infections (NCT03363841).

Rezafungin (CD101, Cidara Therapeutics, San Diego, CA, USA) is an echinocandin that is notable for its long half-life (80 h), allowing once weekly dosing. Potential advantages include simpler management of patients requiring long courses of treatment as outpatients. A phase 2 study is underway, comparing rezafungin with caspofungin followed by fluconazole in patients with invasive candidiasis (NCT02734862) [115].

Oteseconazole (VT-1161, Viamet Pharmaceuticals, San Francisco, CA, USA) is a next-generation azole (tetrazole) with improved specific affinity towards fungal versus mammalian CYP51. As a consequence, VT-1161 is active against fluconazole-resistant *C. glabrata* strains [116,117], and has fewer off-target effects and drug-drug interactions compared with other azoles. It is currently being evaluated in patients with recurrent vulvovaginal candidiasis.

APX001 (Amplyx Pharmaceuticals, San Diego, CA, USA), another first-in-class drug, is a small molecule that inactivates the fungal enzyme Gwt1. This prevents post-translational modification of glycosylphosphatidylinositol (GPI) and disrupts the anchoring of mannoproteins to the fungal cell wall, causing cell wall damage and impaired growth and biofilm formation. APX001 was shown to be active against *C. auris* in vitro, and increased the survival of *C. auris* infected mice (80% to 100% survival versus 50% survival with anidulafungin) [118]. Intravenous and oral formulations are available. Clinical trials are underway.

MAT2203 (Matinas Biopharma, Bedminster, NJ, USA) is a novel encochleated amphotericin B formulation that allows oral administration. Orally administered MAT2203 prevented death and reduced tissue fungal burden in mice with invasive candidiasis [119,120]. Clinical patient data have not yet been published.

## 9. Summary

Treatment of invasive candidiasis is changing. Sensitive molecular diagnostics have the potential to shift treatment strategies from current, risk-based empirical therapy to early targeted treatments. Echinocandins have transformed the care of patients with invasive candidiasis, but their widespread use is leading to rising rates of resistance in *Candida* isolates at some hospitals. Treatment options for drug-resistant *C. glabrata* and *C. auris* are poorly defined, and may well change following results from ongoing clinical trials with novel antifungal agents.

## References

[1] Magill S.S., Edwards J.R., Bamberg W., Beldavs Z.G., Dumyati G., Kainer M.A., Lynfield R., Maloney M., McAllister-Hollod L., Nadle J. (2014). Multistate point-prevalence survey of health care-associated infections. N. Engl. J. Med..

[2] Wisplinghoff H., Bischoff T., Tallent S.M., Seifert H., Wenzel R.P., Edmond M.B. (2004). Nosocomial bloodstream infections in US hospitals: Analysis of 24,179 cases from a prospective nationwide surveillance study. Clin. Infect. Dis..

[3] Vincent J.L., Rello J., Marshall J., Silva E., Anzueto A., Martin C.D., Moreno R., Lipman J., Gomersall C., Sakr Y. (2009). International study of the prevalence and outcomes of infection in intensive care units. JAMA.

[4] Bongomin F., Gago S., Oladele R.O., Denning D.W. (2017). Global and Multi-National Prevalence of Fungal Diseases-Estimate Precision. J. Fungi (Basel).

[5] Cheng S., Clancy C.J., Hartman D.J., Hao B., Nguyen M.H. (2014). *Candida glabrata* Intra-Abdominal Candidiasis Is Characterized by Persistence within the Peritoneal Cavity and Abscesses. Infect. Immun..

[6] Leroy O., Gangneux J.P., Montravers P., Mira J.P., Gouin F., Sollet J.P., Carlet J., Reynes J., Rosenheim M., Regnier B. (2009). Epidemiology, management, and risk factors for death of invasive Candida infections in critical care: A multicenter, prospective, observational study in France (2005–2006). Crit. Care Med..

[7] Strollo S., Lionakis M.S., Adjemian J., Steiner C.A., Prevots D.R. (2016). Epidemiology of Hospitalizations Associated with Invasive Candidiasis, United States, 2002–2012(1). Emerg. Infect. Dis..

[8] Pfaller M.A., Diekema D.J., Gibbs D.L., Newell V.A., Barton R., Bijie H., Bille J., Chang S.C., da Luz Martins M., Duse A. (2010). Geographic variation in the frequency of isolation and fluconazole and voriconazole susceptibilities of *Candida glabrata*: An assessment from the ARTEMIS DISK Global Antifungal Surveillance Program. Diagn. Microbiol. Infect. Dis..

[9] Arendrup M.C. (2010). Epidemiology of invasive candidiasis. Curr. Opin. Crit. Care.

[10] Cleveland A.A., Harrison L.H., Farley M.M., Hollick R., Stein B., Chiller T.M., Lockhart S.R., Park B.J. (2015). Declining incidence of candidemia and the shifting epidemiology of Candida resistance in two US metropolitan areas, 2008–2013: Results from population-based surveillance. PLoS ONE.

[11] Cornely O.A., Bassetti M., Calandra T., Garbino J., Kullberg B.J., Lortholary O., Meersseman W., Akova M., Arendrup M.C., Arikan-Akdagli S. (2012). ESCMID* guideline for the diagnosis and management of *Candida* diseases 2012: Non-neutropenic adult patients. Clin. Microbiol. Infect..

[12] Pappas P.G., Kauffman C.A., Andes D.R., Clancy C.J., Marr K.A., Ostrosky-Zeichner L., Reboli A.C., Schuster M.G., Vazquez J.A., Walsh T.J. (2016). Clinical Practice Guideline for the Management of Candidiasis: 2016 Update by the Infectious Diseases Society of America. Clin. Infect. Dis..

[13] Ullmann A.J., Akova M., Herbrecht R., Viscoli C., Arendrup M.C., Arikan-Akdagli S., Bassetti M., Bille J., Calandra T., Castagnola E. (2012). ESCMID* guideline for the diagnosis and management of Candida diseases 2012: Adults with haematological malignancies and after haematopoietic stem cell transplantation (HCT). Clin. Microbiol. Infect..

[14] Alexander B.D., Johnson M.D., Pfeiffer C.D., Jimenez-Ortigosa C., Catania J., Booker R., Castanheira M., Messer S.A., Perlin D.S., Pfaller M.A. (2013). Increasing echinocandin resistance in *Candida glabrata*: Clinical failure correlates with presence of FKS mutations and elevated minimum inhibitory concentrations. Clin. Infect. Dis..

[15] Ku T.S.N., Walraven C.J., Lee S.A. (2018). *Candida auris*: Disinfectants and Implications for Infection Control. Front. Microbiol..

[16] Lockhart S.R., Etienne K.A., Vallabhaneni S., Farooqi J., Chowdhary A., Govender N.P., Colombo A.L., Calvo B., Cuomo C.A., Desjardins C.A. (2016). Simultaneous Emergence of Multidrug-Resistant *Candida auris* on 3 Continents Confirmed by Whole-Genome Sequencing and Epidemiological Analyses. Clin. Infect. Dis..

[17] Nguyen M.H., Yu V.L. (1995). Meningitis caused by *Candida* species: An emerging problem in neurosurgical patients. Clin. Infect. Dis..

[18] Steinbach W.J., Perfect J.R., Cabell C.H., Fowler V.G., Corey G.R., Li J.S., Zaas A.K., Benjamin D.K. (2005). A meta-analysis of medical versus surgical therapy for Candida endocarditis. J. Infect..

[19] Garcia-Effron G., Katiyar S.K., Park S., Edlind T.D., Perlin D.S. (2008). A naturally occurring proline-to-alanine amino acid change in Fks1p in *Candida parapsilosis*, Candida orthopsilosis, and Candida metapsilosis accounts for reduced echinocandin susceptibility. Antimicrob. Agents Chemother..

[20] Mora-Duarte J., Betts R., Rotstein C., Colombo A.L., Thompson-Moya L., Smietana J., Lupinacci R., Sable C., Kartsonis N., Perfect J. (2002). Comparison of caspofungin and amphotericin B for invasive candidiasis. N. Engl. J. Med..

[21] Kuse E.R., Chetchotisakd P., da Cunha C.A., Ruhnke M., Barrios C., Raghunadharao D., Sekhon J.S., Freire A., Ramasubramanian V., Demeyer I. (2007). Micafungin versus liposomal amphotericin B for candidaemia and invasive candidosis: A phase III randomised double-blind trial. Lancet.

[22] Reboli A.C., Rotstein C., Pappas P.G., Chapman S.W., Kett D.H., Kumar D., Betts R., Wible M., Goldstein B.P., Schranz J. (2007). Anidulafungin versus fluconazole for invasive candidiasis. N. Engl. J. Med..

[23] Pappas P.G., Rotstein C.M., Betts R.F., Nucci M., Talwar D., De Waele J.J., Vazquez J.A., Dupont B.F., Horn D.L., Ostrosky-Zeichner L. (2007). Micafungin versus caspofungin for treatment of candidemia and other forms of invasive candidiasis. Clin. Infect. Dis..

[24] Betts R.F., Nucci M., Talwar D., Gareca M., Queiroz-Telles F., Bedimo R.J., Herbrecht R., Ruiz-Palacios G., Young J.A., Baddley J.W. (2009). A Multicenter, double-blind trial of a high-dose caspofungin treatment regimen versus a standard caspofungin treatment regimen for adult patients with invasive candidiasis. Clin. Infect. Dis..

[25] Hitchcock C.A., Dickinson K., Brown S.B., Evans E.G., Adams D.J. (1990). Interaction of azole antifungal antibiotics with cytochrome P-450-dependent 14 alpha-sterol demethylase purified from *Candida albicans*. Biochem. J..

[26] Rex J.H., Bennett J.E., Sugar A.M., Pappas P.G., van der Horst C.M., Edwards J.E., Washburn R.G., Scheld W.M., Karchmer A.W., Dine A.P. (1994). A randomized trial comparing fluconazole with amphotericin B for the treatment of candidemia in patients without neutropenia. Candidemia Study Group and the National Institute. N. Engl. J. Med..

[27] Phillips P., Shafran S., Garber G., Rotstein C., Smaill F., Fong I., Salit I., Miller M., Williams K., Conly J.M. (1997). Multicenter randomized trial of fluconazole versus amphotericin B for treatment of candidemia in non-neutropenic patients. Canadian Candidemia Study Group. Eur. J. Clin. Microbiol. Infect. Dis..

[28] Rex J.H., Pappas P.G., Karchmer A.W., Sobel J., Edwards J.E., Hadley S., Brass C., Vazquez J.A., Chapman S.W., Horowitz H.W. (2003). A randomized and blinded multicenter trial of high-dose fluconazole plus placebo versus fluconazole plus amphotericin B as therapy for candidemia and its consequences in nonneutropenic subjects. Clin. Infect. Dis..

[29] Kullberg B.J., Sobel J.D., Ruhnke M., Pappas P.G., Viscoli C., Rex J.H., Cleary J.D., Rubinstein E., Church L.W.P., Brown J.M. (2005). Voriconazole versus a regimen of amphotericin B followed by fluconazole for candidaemia in non-neutropenic patients: A randomised non-inferiority trial. Lancet.

[30] Bates D.W., Su L., Yu D.T., Chertow G.M., Seger D.L., Gomes D.R., Dasbach E.J., Platt R. (2001). Mortality and costs of acute renal failure associated with amphotericin B therapy. Clin. Infect. Dis..

[31] Clinical and Laboratory Standards Institute (2008). Reference Method for Broth Dilution Antifungal Susceptibility Testing of Yeasts.

[32] EUCAST (2008). Definitive document EDef 7.1: Method for the determination of broth dilution MICs of antifungal agents for fermentative yeasts. Clin. Microbiol. Infect..

[33] Ben-Ami R., Hilerowicz Y., Novikov A., Giladi M. (2014). The impact of new epidemiological cutoff values on *Candida glabrata* resistance rates and concordance between testing methods. Diagn. Microbiol. Infect. Dis..

[34] Shields R.K., Nguyen M.H., Press E.G., Updike C.L., Clancy C.J. (2013). Anidulafungin and micafungin MIC breakpoints are superior to that of caspofungin for identifying FKS mutant *Candida glabrata* strains and Echinocandin resistance. Antimicrob. Agents Chemother..

[35] Eschenauer G.A., Nguyen M.H., Shoham S., Vazquez J.A., Morris A.J., Pasculle W.A., Kubin C.J., Klinker K.P., Carver P.L., Hanson K.E. (2014). Real-world experience with echinocandin MICs against *Candida* species in a multicenter study of hospitals that routinely perform susceptibility testing of bloodstream isolates. Antimicrob. Agents Chemother..

[36] Arendrup M.C., Pfaller M.A., Danish Fungaemia Study Group (2012). Caspofungin Etest susceptibility testing of *Candida* species: Risk of misclassification of susceptible isolates of *C. glabrata* and *C. krusei* when adopting the revised CLSI caspofungin breakpoints. Antimicrob. Agents Chemother..

[37] Chamilos G., Lewis R.E., Albert N., Kontoyiannis D.P. (2007). Paradoxical effect of Echinocandins across *Candida* species in vitro: Evidence for echinocandin-specific and candida species-related differences. Antimicrob. Agents Chemother..

[38] Fleischhacker M., Radecke C., Schulz B., Ruhnke M. (2008). Paradoxical growth effects of the echinocandins caspofungin and micafungin, but not of anidulafungin, on clinical isolates of *Candida albicans* and *C. dubliniensis*. Eur. J. Clin. Microbiol. Infect. Dis..

[39] Shields R.K., Nguyen M.H., Du C., Press E., Cheng S., Clancy C.J. (2011). Paradoxical effect of caspofungin against *Candida* bloodstream isolates is mediated by multiple pathways but eliminated in human serum. Antimicrob. Agents Chemother..

[40] Bayegan S., Majoros L., Kardos G., Kemeny-Beke A., Miszti C., Kovacs R., Gesztelyi R. (2010). In vivo studies with a Candida tropicalis isolate exhibiting paradoxical growth in vitro in the presence of high concentration of caspofungin. J. Microbiol..

[41] Shields R.K., Nguyen M.H., Press E.G., Kwa A.L., Cheng S., Du C., Clancy C.J. (2012). The presence of an FKS mutation rather than MIC is an independent risk factor for failure of echinocandin therapy among patients with invasive candidiasis due to *Candida glabrata*. Antimicrob. Agents Chemother..

[42] Shields R.K., Nguyen M.H., Press E.G., Updike C.L., Clancy C.J. (2013). Caspofungin MICs correlate with treatment outcomes among patients with *Candida glabrata* invasive candidiasis and prior echinocandin exposure. Antimicrob. Agents Chemother..

[43] Ben-Ami R., Garcia-Effron G., Lewis R.E., Gamarra S., Leventakos K., Perlin D.S., Kontoyiannis D.P. (2011). Fitness and Virulence Costs of *Candida albicans* FKS1 Hot Spot Mutations Associated With Echinocandin Resistance. J. Infect. Dis..

[44] Singh-Babak S.D., Babak T., Diezmann S., Hill J.A., Xie J.L., Chen Y.L., Poutanen S.M., Rennie R.P., Heitman J., Cowen L.E. (2012). Global Analysis of the Evolution and Mechanism of Echinocandin Resistance in *Candida glabrata*. PLoS Pathog..

[45] Dannaoui E., Desnos-Ollivier M., Garcia-Hermoso D., Grenouillet F., Cassaing S., Baixench M.T., Bretagne S., Dromer F., Lortholary O., French Mycoses Study Group (2012). *Candida* spp. with acquired echinocandin resistance, France, 2004-2010. Emerg. Infect. Dis..

[46] Rex J.H., Pfaller M.A. (2002). Has antifungal susceptibility testing come of age?. Clin. Infect. Dis..

[47] Pfaller M.A., Andes D., Diekema D.J., Espinel-Ingroff A., Sheehan D. (2010). Wild-type MIC distributions, epidemiological cutoff values and species-specific clinical breakpoints for fluconazole and *Candida*: Time for harmonization of CLSI and EUCAST broth microdilution methods. Drug. Resist. Updat..

[48] Izumikawa K., Kakeya H., Tsai H.F., Grimberg B., Bennett J.E. (2003). Function of *Candida glabrata* ABC transporter gene, *PDH1*. Yeast.

[49] White T.C. (1997). Increased mRNA levels of ERG16, CDR, and MDR1 correlate with increases in azole resistance in *Candida albicans* isolates from a patient infected with human immunodeficiency virus. Antimicrob. Agents Chemother..

[50] Flowers S.A., Barker K.S., Berkow E.L., Toner G., Chadwick S.G., Gygax S.E., Morschhauser J., Rogers P.D. (2012). Gain-of-function mutations in UPC2 are a frequent cause of ERG11 upregulation in azole-resistant clinical isolates of *Candida albicans*. Eukaryot Cell.

[51] Sanglard D., Ischer F., Calabrese D., Majcherczyk P.A., Bille J. (1999). The ATP binding cassette transporter gene *CgCDR1* from *Candida glabrata* is involved in the resistance of clinical isolates to azole antifungal agents. Antimicrob. Agents Chemother..

[52] Miyazaki H., Miyazaki Y., Geber A., Parkinson T., Hitchcock C., Falconer D.J., Ward D.J., Marsden K., Bennett J.E. (1998). Fluconazole resistance associated with drug efflux and increased transcription of a drug transporter gene, *PDH1*, in *Candida glabrata*. Antimicrob. Agents Chemother..

[53] Vermitsky J.-P., Earhart K.D., Smith W.L., Homayouni R., Edlind T.D., Rogers P.D. (2006). Pdr1 regulates multidrug resistance in *Candida glabrata*: Gene disruption and genome-wide expression studies. Mol. Microbiol..

[54] Vermitsky J.P., Edlind T.D. (2004). Azole resistance in *Candida glabrata*: Coordinate upregulation of multidrug transporters and evidence for a Pdr1-like transcription factor. Antimicrob. Agents Chemother..

[55] Ferrari S., Ischer F., Calabrese D., Posteraro B., Sanguinetti M., Fadda G., Rohde B., Bauser C., Bader O., Sanglard D. (2009). Gain of function mutations in CgPDR1 of *Candida glabrata* not only mediate antifungal resistance but also enhance virulence. PLoS Pathog..

[56] Tsai H.F., Krol A.A., Sarti K.E., Bennett J.E. (2006). *Candida glabrata PDR1*, a transcriptional regulator of a pleiotropic drug resistance network, mediates azole resistance in clinical isolates and petite mutants. Antimicrob. Agents Chemother..

[57] Bennett J.E., Izumikawa K., Marr K.A. (2004). Mechanism of increased fluconazole resistance in *Candida glabrata* during prophylaxis. Antimicrob. Agents Chemother..

[58] Ben-Ami R., Berman J., Novikov A., Bash E., Shachor-Meyouhas Y., Zakin S., Maor Y., Tarabia J., Schechner V., Adler A. (2017). Multidrug-Resistant *Candida haemulonii* and *C. auris*, Tel Aviv, Israel. Emerg. Infect. Dis..

[59] Chowdhary A., Sharma C., Meis J.F. (2017). *Candida auris*: A rapidly emerging cause of hospital-acquired multidrug-resistant fungal infections globally. PLoS Pathog..

[60] Park B.J., Arthington-Skaggs B.A., Hajjeh R.A., Iqbal N., Ciblak M.A., Lee-Yang W., Hairston M.D., Phelan M., Plikaytis B.D., Sofair A.N. (2006). Evaluation of amphotericin B interpretive breakpoints for *Candida* bloodstream isolates by correlation with therapeutic outcome. Antimicrob. Agents Chemother..

[61] Nguyen M.H., Clancy C.J., Yu V.L., Yu Y.C., Morris A.J., Snydman D.R., Sutton D.A., Rinaldi M.G. (1998). Do in vitro susceptibility data predict the microbiologic response to amphotericin B? Results of a prospective study of patients with Candida fungemia. J. Infect. Dis..

[62] Lass-Florl C., Arendrup M.C., Rodriguez-Tudela J.L., Cuenca-Estrella M., Donnelly P., Hope W., European Committee on Antimicrobial Susceptibility Testing—Subcommittee on Antifungal Susceptibility Testing (2011). EUCAST technical note on Amphotericin B. Clin. Microbiol. Infect..

[63] Martel C.M., Parker J.E., Bader O., Weig M., Gross U., Warrilow A.G., Rolley N., Kelly D.E., Kelly S.L. (2010). Identification and characterization of four azole-resistant erg3 mutants of *Candida albicans*. Antimicrob. Agents Chemother..

[64] Martel C.M., Parker J.E., Bader O., Weig M., Gross U., Warrilow A.G., Kelly D.E., Kelly S.L. (2010). A clinical isolate of *Candida albicans* with mutations in ERG11 (encoding sterol 14alpha-demethylase) and ERG5 (encoding C22 desaturase) is cross resistant to azoles and amphotericin B. Antimicrob. Agents Chemother..

[65] Sanglard D., Ischer F., Parkinson T., Falconer D., Bille J. (2003). *Candida albicans* mutations in the ergosterol biosynthetic pathway and resistance to several antifungal agents. Antimicrob. Agents Chemother..

[66] Jensen R.H., Astvad K.M., Silva L.V., Sanglard D., Jorgensen R., Nielsen K.F., Mathiasen E.G., Doroudian G., Perlin D.S., Arendrup M.C. (2015). Stepwise emergence of azole, echinocandin and amphotericin B multidrug resistance in vivo in *Candida albicans* orchestrated by multiple genetic alterations. J. Antimicrob. Chemother..

[67] Healey K.R., Zhao Y., Perez W.B., Lockhart S.R., Sobel J.D., Farmakiotis D., Kontoyiannis D.P., Sanglard D., Taj-Aldeen S.J., Alexander B.D. (2016). Prevalent mutator genotype identified in fungal pathogen *Candida glabrata* promotes multi-drug resistance. Nat. Commun..

[68] Ben-Ami R., Zimmerman O., Finn T., Amit S., Novikov A., Wertheimer N., Lurie-Weinberger M., Berman J. (2016). Heteroresistance to Fluconazole Is a Continuously Distributed Phenotype among *Candida glabrata* Clinical Strains Associated with In Vivo Persistence. mBio.

[69] El-Halfawy O.M., Valvano M.A. (2015). Antimicrobial heteroresistance: An emerging field in need of clarity. Clin. Microbiol. Rev..

[70] Morrell M., Fraser V.J., Kollef M.H. (2005). Delaying the empiric treatment of candida bloodstream infection until positive blood culture results are obtained: A potential risk factor for hospital mortality. Antimicrob. Agents Chemother..

[71] Garey K.W., Rege M., Pai M.P., Mingo D.E., Suda K.J., Turpin R.S., Bearden D.T. (2006). Time to initiation of fluconazole therapy impacts mortality in patients with candidemia: A multi-institutional study. Clin. Infect. Dis..

[72] Farmakiotis D., Kyvernitakis A., Tarrand J.J., Kontoyiannis D.P. (2015). Early initiation of appropriate treatment is associated with increased survival in cancer patients with *Candida glabrata* fungaemia: A potential benefit from infectious disease consultation. Clin. Microbiol. Infect..

[73] Kollef M., Micek S., Hampton N., Doherty J.A., Kumar A. (2012). Septic shock attributed to Candida infection: Importance of empiric therapy and source control. Clin. Infect. Dis..

[74] Clancy C.J., Nguyen M.H. (2013). Finding the “Missing 50%” of Invasive Candidiasis: How Nonculture Diagnostics Will Improve Understanding of Disease Spectrum and Transform Patient Care. Clin. Infect. Dis..

[75] Pfeiffer C.D., Samsa G.P., Schell W.A., Reller L.B., Perfect J.R., Alexander B.D. (2011). Quantitation of Candida CFU in initial positive blood cultures. J. Clin. Microbiol..

[76] Leon C., Ruiz-Santana S., Saavedra P., Almirante B., Nolla-Salas J., Alvarez-Lerma F., Garnacho-Montero J., Leon M.A. (2006). A bedside scoring system (“*Candida* score”) for early antifungal treatment in nonneutropenic critically ill patients with *Candida* colonization. Crit. Care Med..

[77] Bruyere R., Quenot J.P., Prin S., Dalle F., Vigneron C., Aho S., Leon C., Charles P.E. (2014). Empirical antifungal therapy with an echinocandin in critically-ill patients: Prospective evaluation of a pragmatic *Candida* score-based strategy in one medical ICU. BMC Infect. Dis..

[78] Posteraro B., De Pascale G., Tumbarello M., Torelli R., Pennisi M.A., Bello G., Maviglia R., Fadda G., Sanguinetti M., Antonelli M. (2011). Early diagnosis of candidemia in intensive care unit patients with sepsis: A prospective comparison of (1-->3)-beta-d-glucan assay, *Candida* score, and colonization index. Crit. Care.

[79] Leroy G., Lambiotte F., Thevenin D., Lemaire C., Parmentier E., Devos P., Leroy O. (2011). Evaluation of “*Candida* score” in critically ill patients: A prospective, multicenter, observational, cohort study. Ann. Intensive Care.

[80] Karageorgopoulos D.E., Vouloumanou E.K., Ntziora F., Michalopoulos A., Rafailidis P.I., Falagas M.E. (2011). beta-D-glucan assay for the diagnosis of invasive fungal infections: A meta-analysis. Clin. Infect. Dis..

[81] Mikulska M., Calandra T., Sanguinetti M., Poulain D., Viscoli C., Third European Conference on Infections in Leukemia Group (2010). The use of mannan antigen and anti-mannan antibodies in the diagnosis of invasive candidiasis: Recommendations from the Third European Conference on Infections in Leukemia. Crit. Care.

[82] Avni T., Leibovici L., Paul M. (2011). PCR diagnosis of invasive candidiasis: Systematic review and meta-analysis. J. Clin. Microbiol..

[83] Clancy C.J., Pappas P.G., Vazquez J., Judson M.A., Kontoyiannis D.P., Thompson G.R., Garey K.W., Reboli A., Greenberg R.N., Apewokin S. (2018). Detecting Infections Rapidly and Easily for Candidemia Trial, Part 2 (DIRECT2): A Prospective, Multicenter Study of the T2Candida Panel. Clin. Infect. Dis..

[84] Andes D.R., Safdar N., Baddley J.W., Playford G., Reboli A.C., Rex J.H., Sobel J.D., Pappas P.G., Kullberg B.J. (2012). Impact of treatment strategy on outcomes in patients with candidemia and other forms of invasive candidiasis: A patient-level quantitative review of randomized trials. Clin. Infect. Dis..

[85] Lortholary O., Desnos-Ollivier M., Sitbon K., Fontanet A., Bretagne S., Dromer F., French Mycosis Study Group (2011). Recent exposure to caspofungin or fluconazole influences the epidemiology of candidemia: A prospective multicenter study involving 2441 patients. Antimicrob. Agents Chemother..

[86] Forrest G.N., Weekes E., Johnson J.K. (2008). Increasing incidence of *Candida parapsilosis* candidemia with caspofungin usage. J. Infect..

[87] Sipsas N.V., Lewis R.E., Tarrand J., Hachem R., Rolston K.V., Raad I.I., Kontoyiannis D.P. (2009). Candidemia in patients with hematologic malignancies in the era of new antifungal agents (2001–2007): Stable incidence but changing epidemiology of a still frequently lethal infection. Cancer.

[88] Fernandez-Ruiz M., Aguado J.M., Almirante B., Lora-Pablos D., Padilla B., Puig-Asensio M., Montejo M., Garcia-Rodriguez J., Peman J., Ruiz Perez de Pipaon M. (2014). Initial use of echinocandins does not negatively influence outcome in *Candida parapsilosis* bloodstream infection: A propensity score analysis. Clin. Infect. Dis..

[89] Centers for Disease Control Recommendations for Treatment of Candida auris. https://www.cdc.gov/fungal/candida-auris/c-auris-treatment.html.

[90] Ruiz-Gaitan A., Moret A.M., Tasias-Pitarch M., Aleixandre-Lopez A.I., Martinez-Morel H., Calabuig E., Salavert-Lleti M., Ramirez P., Lopez-Hontangas J.L., Hagen F. (2018). An outbreak due to *Candida auris* with prolonged colonization and candidemia in a tertiary care European hospital. Mycoses.

[91] Rudramurthy S.M., Chakrabarti A., Paul R.A., Sood P., Kaur H., Capoor M.R., Kindo A.J., Marak R.S., Arora A., Sardana R. (2017). *Candida auris* candidaemia in Indian ICUs: Analysis of risk factors. J. Antimicrob. Chemother..

[92] Ben-Ami R., Novikov A., Koralker N., Berman J., Ashkenazi L. (2017). Assessment of *Candida auris* response to antifungal drugs using time-kill assays and an animal model. Open Forum Infect. Dis..

[93] Oude Lashof A.M., Donnelly J.P., Meis J.F., van der Meer J.W., Kullberg B.J. (2003). Duration of antifungal treatment and development of delayed complications in patients with candidaemia. Eur. J. Clin. Microbiol. Infect. Dis..

[94] Vazquez J., Reboli A.C., Pappas P.G., Patterson T.F., Reinhardt J., Chin-Hong P., Tobin E., Kett D.H., Biswas P., Swanson R. (2014). Evaluation of an early step-down strategy from intravenous anidulafungin to oral azole therapy for the treatment of candidemia and other forms of invasive candidiasis: Results from an open-label trial. BMC Infect. Dis..

[95] Pfaller M., Neofytos D., Diekema D., Azie N., Meier-Kriesche H.U., Quan S.P., Horn D. (2012). Epidemiology and outcomes of candidemia in 3648 patients: Data from the Prospective Antifungal Therapy (PATH Alliance(R)) registry, 2004–2008. Diagn. Microbiol. Infect. Dis..

[96] Liu C.Y., Huang L.J., Wang W.S., Chen T.L., Yen C.C., Yang M.H., Hsiao L.T., Liu C.Y., Chen P.M., Chiou T.J. (2009). Candidemia in cancer patients: Impact of early removal of non-tunneled central venous catheters on outcome. J. Infect..

[97] Nguyen M.H., Peacock J.E., Tanner D.C., Morris A.J., Nguyen M.L., Snydman D.R., Wagener M.M., Yu V.L. (1995). Therapeutic approaches in patients with candidemia. Evaluation in a multicenter, prospective, observational study. Arch. Intern. Med..

[98] Nucci M., Anaissie E., Betts R.F., Dupont B.F., Wu C., Buell D.N., Kovanda L., Lortholary O. (2010). Early removal of central venous catheter in patients with candidemia does not improve outcome: Analysis of 842 patients from 2 randomized clinical trials. Clin. Infect. Dis..

[99] Park K.H., Lee M.S., Lee S.O., Choi S.H., Sung H., Kim M.N., Kim Y.S., Woo J.H., Kim S.H. (2014). Diagnostic usefulness of differential time to positivity for catheter-related candidemia. J. Clin. Microbiol..

[100] Ben-Ami R., Weinberger M., Orni-Wasserlauff R., Schwartz D., Itzhaki A., Lazarovitch T., Bash E., Aharoni Y., Moroz I., Giladi M. (2008). Time to blood culture positivity as a marker for catheter-related candidemia. J. Clin. Microbiol..

[101] Stempel J.M., Farmakiotis D., Tarrand J.J., Kontoyiannis D.P. (2016). Time-to-reporting of blood culture positivity and central venous catheter-associated *Candida glabrata* fungemia in cancer patients. Diagn Microbiol. Infect. Dis..

[102] Mermel Leonard A., Allon M., Bouza E., Craven D.E., Flynn P., O’Grady N.P., Raad I.I., Rijnders B.J.A., Sherertz R.J., Warren D.K. (2009). Clinical Practice Guidelines for the Diagnosis and Management of Intravascular Catheter-Related Infection: 2009 Update by the Infectious Diseases Society of America. Clin. Infect. Dis..

[103] Walraven C.J., Lee S.A. (2013). Antifungal lock therapy. Antimicrob. Agents Chemother..

[104] Shields R.K., Nguyen M.H., Press E.G., Clancy C.J. (2014). Abdominal candidiasis is a hidden reservoir of echinocandin resistance. Antimicrob. Agents Chemother..

[105] Fernandez-Cruz A., Cruz Menarguez M., Munoz P., Pedromingo M., Pelaez T., Solis J., Rodriguez-Creixems M., Bouza E., Group G.S. (2015). The search for endocarditis in patients with candidemia: A systematic recommendation for echocardiography? A prospective cohort. Eur. J. Clin. Microbiol. Infect. Dis..

[106] Oude Lashof A.M., Rothova A., Sobel J.D., Ruhnke M., Pappas P.G., Viscoli C., Schlamm H.T., Oborska I.T., Rex J.H., Kullberg B.J. (2011). Ocular manifestations of candidemia. Clin. Infect. Dis..

[107] Donahue S.P., Greven C.M., Zuravleff J.J., Eller A.W., Nguyen M.H., Peacock J.E., Wagener M.W., Yu V.L. (1994). Intraocular candidiasis in patients with candidemia. Clinical implications derived from a prospective multicenter study. Ophthalmology.

[108] Akler M.E., Vellend H., McNeely D.M., Walmsley S.L., Gold W.L. (1995). Use of fluconazole in the treatment of candidal endophthalmitis. Clin. Infect. Dis..

[109] Mochizuki K., Sawada A., Suemori S., Kawakami H., Niwa Y., Kondo Y., Ohkusu K., Yamada N., Ogura S., Yaguchi T. (2013). Intraocular penetration of intravenous micafungin in inflamed human eyes. Antimicrob. Agents Chemother..

[110] Gauthier G.M., Nork T.M., Prince R., Andes D. (2005). Subtherapeutic ocular penetration of caspofungin and associated treatment failure in *Candida albicans* endophthalmitis. Clin. Infect. Dis..

[111] Schell W.A., Jones A.M., Borroto-Esoda K., Alexander B.D. (2017). Antifungal Activity of SCY-078 and Standard Antifungal Agents against 178 Clinical Isolates of Resistant and Susceptible *Candida* Species. Antimicrob. Agents Chemother..

[112] Larkin E., Hager C., Chandra J., Mukherjee P.K., Retuerto M., Salem I., Long L., Isham N., Kovanda L., Borroto-Esoda K. (2017). The Emerging Pathogen *Candida auris*: Growth Phenotype, Virulence Factors, Activity of Antifungals, and Effect of SCY-078, a Novel Glucan Synthesis Inhibitor, on Growth Morphology and Biofilm Formation. Antimicrob. Agents Chemother..

[113] Wiederhold N.P., Najvar L.K., Jaramillo R., Olivo M., Pizzini J., Catano G., Patterson T.F. (2018). Oral glucan synthase inhibitor SCY-078 is effective in an experimental murine model of invasive candidiasis caused by WT and echinocandin-resistant *Candida glabrata*. J. Antimicrob. Chemother..

[114] Jimenez-Ortigosa C., Perez W.B., Angulo D., Borroto-Esoda K., Perlin D.S. (2017). De Novo Acquisition of Resistance to SCY-078 in *Candida glabrata* Involves FKS Mutations That both Overlap and Are Distinct from Those Conferring Echinocandin Resistance. Antimicrob. Agents Chemother..

[115] Sofjan A.K., Mitchell A., Shah D.N., Nguyen T., Sim M., Trojcak A., Beyda N.D., Garey K.W. (2018). Rezafungin (CD101), a next-generation echinocandin: A systematic literature review and assessment of possible place in therapy. J. Glob. Antimicrob. Resist..

[116] Brand S.R., Degenhardt T.P., Person K., Sobel J.D., Nyirjesy P., Schotzinger R.J., Tavakkol A. (2018). A phase 2, randomized, double-blind, placebo-controlled, dose-ranging study to evaluate the efficacy and safety of orally administered VT-1161 in the treatment of recurrent vulvovaginal candidiasis. Am. J. Obstet. Gynecol..

[117] Break T.J., Desai J.V., Natarajan M., Ferre E.M.N., Henderson C., Zelazny A.M., Siebenlist U., Hoekstra W.J., Schotzinger R.J., Garvey E.P. (2018). VT-1161 protects mice against oropharyngeal candidiasis caused by fluconazole-susceptible and -resistant *Candida albicans*. J. Antimicrob. Chemother..

[118] Hager C.L., Larkin E.L., Long L., Zohra Abidi F., Shaw K.J., Ghannoum M.A. (2018). In Vitro and In Vivo Evaluation of the Antifungal Activity of APX001A/APX001 against *Candida auris*. Antimicrob. Agents Chemother..

[119] Santangelo R., Paderu P., Delmas G., Chen Z.W., Mannino R., Zarif L., Perlin D.S. (2000). Efficacy of oral cochleate-amphotericin B in a mouse model of systemic candidiasis. Antimicrob. Agents Chemother..

[120] Zarif L., Graybill J.R., Perlin D., Najvar L., Bocanegra R., Mannino R.J. (2000). Antifungal activity of amphotericin B cochleates against *Candida albicans* infection in a mouse model. Antimicrob. Agents Chemother..

